# The Cox-2 Inhibitor Meloxicam Ameliorates Neuroinflammation and Depressive Behavior in Adult Mice after Splenectomy

**Published:** 2016-08-05

**Authors:** Michael Haile, Allal Boutajangout, Kevin Chung, Jeffrey Chan, Tanya Stolper, Nemahun Vincent, Marc Batchan, John D’Urso, Yan Lin, Richard Kline, Faris Yaghmoor, Saad Jahfal, Robel Kamal, Waleed Aljohani, Thomas Blanck, Alex Bekker, Thomas Wisniewski

**Affiliations:** 1 Department of Anesthesiology, New York University School of Medicine; 2 Department of Neurology, New York University School of Medicine; 3 Department of Psychiatry, New York University School of Medicine; 4 Department of Pathology, New York University School of Medicine; 5 Department of Physiology, Neuroscience, New York, New York; 6 Rutgers New Jersey Medical School, New York University School of Medicine, New Jersey; 7 King Abdulaziz University, Jeddah, Saudi Arabia

**Keywords:** Anhedonia, Depression, Neuroinflammation, Meloxicam, Cyclooxygenase-2, Splenectomy, Astrocytosis, Microgliosis

## Abstract

**Background:**

Peripheral surgical trauma may incite neuroinflammation that leads to neuronal dysfunction associated with both depression and cognitive deficits. In a previous study, we found that adult mice developed neuroinflammation and short-term working memory dysfunction in a delayed, transient manner after splenectomy that was ameliorated by the cyclooxygenase-2 inhibitor meloxicam. We tested the hypothesis that splenectomy in mice would also cause anhedonia, the diminished response to pleasure or rewarding stimuli that is a hallmark of depression, and that treatment with meloxicam would be ameliorative.

**Methods:**

After Institutional Animal Care and Use Committee approval, Swiss-Webster mice underwent sucrose preference training before being randomized into groups on day 0, when they had either splenectomy and anesthesia or anesthesia alone. Within each group, half were randomized to receive intraperitoneal saline at 24 hours, while the other half received intraperitoneal meloxicam at 24 hours. Sucrose preference ratios were determined on days 1, 5, 9, and 14. Additional mice were randomized into groups for brain histochemistry. Specimens were stained for glial fibrillary acidic protein (GFAP), a marker of astrocytes, and CD45, a protein tyrosine phosphatase that identifies microglial activation.

**Results:**

On day 5, mice receiving splenectomy and saline demonstrated diminished sucrose preference, which was not seen in mice receiving splenectomy and meloxicam. Semiquantitative analysis of histological slides taken from splenectomized mice treated with meloxicam revealed reduced microglial-based neuroinflammation and reactive astrocytosis compared to mice receiving saline.

**Conclusion:**

Splenectomy in mice is associated with neuroinflammation and anhedonia, as evidenced by reactive microgliosis, astrocytosis, and behavioral changes. Postsurgical treatment with meloxicam attenuates both neuroinflammation and anhedonia. These findings suggest that cyclooxygenase-2-dependent mechanisms may play a role in the development of postoperative mood disorders, possibly via modulation of peripheral effects on neuroinflammation.

## Background

Peripheral surgical trauma and the ensuing cytokine-mediated inflammatory response can result in central nervous system immune activation which, in turn, leads to neuronal and synaptic dysfunction associated with cognitive deficits and depression [1–4]. Mood disorders are a group of diseases which which have a disturbance of mood as a prominent feature, can also include cognitive, psychomotor and interpersonal psycho-physiological disfunction. Mood disorders like depression often accompany stressful events and may lead to a decline in function and of the quality of life with attendant morbidity and mortality [5,6]. The triad of depressed mood, cognitive deficits, and inflammation has been observed in patients following both cardiac and non-cardiac surgeries and has called postoperative cognitive dysfunction (POCD) [7–9]. Splenectomy has been used as a well characterized animal model of POCD [5,10–14].

In our previous study, we demonstrated that adult mice developed neuroinflammation and short-term working memory dysfunction in a delayed, transient manner after splenectomy. Both inflammation and memory dysfunction were relieved by treatment with the cyclooxygenase-2(COX-2) inhibitor meloxicam(MEL) [10]. In our current study, we tested the hypothesis that splenectomy would also cause depressive behavior in mice and that treatment with MEL would prevent the development of anhedonia, the diminished response to pleasure or rewarding stimuli that is a hallmark of depression.[15]

## Methods

### Ethics Statement

This study was approved by the New York University Langone Medical Center Institutional Animal Care and Use Committee (IACUC). 169 male Swiss Webster mice, 6–8 weeks old and 35–45 grams in weight, (Taconic, Albany, New York, USA) were used for behavioral studies.

### Sucrose Preference Training

Mice were trained for two weeks before surgery using a sucrose preference protocol as previously published [16]. They were kept individually with tap water freely available from two graduated cylinders. Every fourth night, the mice were fluid restricted before being given a choice of either 2% sucrose or water from the cylinders for one hour the following morning. The positions of the cylinder containing sucrose and the cylinder containing water were alternated with each testing and training day. Baseline sucrose preference ratios determined by the ratio of sucrose fluid to total fluid consumed were established.

### Surgery and/or Anesthesia

After sucrose preference training, mice were randomized into two groups on day 0:

1. Splenectomy and anesthesia
2. Anesthesia alone

Splenectomy is a surgical procedure that has been shown to lead to neuroinflammation and cognitive dysfunction in rodents [5,10–14]. Induction of anesthesia was performed with 2.5% isoflurane in 30% oxygen / 70% nitrogen inside an anesthesia chamber (Harvard Apparatus, Holliston MA, USA). Mice randomized to receive splenectomy had a small rodent face mask applied and received 2.5% isoflurane. The abdominal wall was shaved prior to making a 1 cm abdominal incision. Under neurosurgical microscopic guidance, the spleen was freed from the surrounding tissue by blunt dissection. Blood vessels were ligated using 6–0 silk suture, and the spleen was removed by transecting the blood vessels distal to the ligature. The skin incision was closed using 4–0 silk suture. The time of surgery was approximately five minutes. To minimize variability, all surgeries were performed by the same individual. Under identical conditions, mice randomized to receive anesthesia alone were anesthetized on day 0 for the same amount of time without undergoing surgery. For all mice, inspired gas levels were monitored with a gas analyzer (Capnomac Ultima, Datex Ohmeda, Helsinki, Finland), and a thermal blanket was used to monitor and maintain a rectal temperature of 37 ± 0.5 °C.

### Treatment

The two groups of mice were further randomized into two groups each, resulting in four groups:

1. Splenectomy with anesthesia followed by intraperitoneal (i.p.) saline (SAL) at 24 hours
2. Splenectomy with anesthesia followed by i.p. MEL at 24 hours
3. Anesthesia alone followed by i.p. SAL at 24 hours
4. Anesthesia alone followed by i.p. MEL at 24 hours

Groups 1 and 3 received i.p. SAL (0.9% NaCl in500 µL) 24 hours after splenectomy and/or anesthesia.

Groups 2 and 4 received i.p. MEL (60 mg/kg in 500 µL saline) 24 hours after splenectomy and/or anesthesia.

### Sucrose Preference Testing

All mice underwent testing for sucrose preference ratios over an additional two weeks. Following surgery and/or anesthesia, mice were kept as they had been during the sucrose preference training period. Every fourth day, mice were water deprived overnight and on days 1, 5, 9, and 14, sucrose preference ratios were determined by the ratio of sucrose fluid to total fluid consumed.

### Locomotor Testing

Ten additional Swiss-Webster, 30–40 gram mice (6–8 weeks old) were tested on the running wheel to measure locomotive behavior over the time course of the study. For three days, mice were housed singly in cages (22 × 22 × 46 cm) equipped with a running wheel (18 cm diameter, 8 cm wide) to establish a baseline. Mice then had splenectomy with anesthesia or received anesthesia alone as above. At 24 hours, mice then received either i.p. MEL (n=4) or SAL (n=5). For 12 days after injection, each mouse remained in its cage with continued daily recording of the number of revolutions of the wheel caused by running. The average number of revolutions per hour was calculated as the relative measure of locomotion.

### Histochemistry

Thirty five additional mice had splenectomy performed as above and were randomized into seven groups of five mice each. Mice that were not sacrificed on day 1 for histochemistry received either i.p. MEL or SAL at 24 hours before being sacrificed on the day indicated.

1. Day 1
2. MEL then Day 5
3. SAL then Day 5
4. SAL then Day 9
5. MEL then Day 9
6. SAL then Day 14
7. MEL then Day 14

Before sacrifice, mice were anesthetized with ketamine (150 mg kg) and xylazine (10 mg kg) using i.p. injections and perfused transaortically with phosphate buffer solution (PBS) containing heparin (APP Pharmaceuticals, IL, USA) followed by 4% paraformaldehyde (Fisher Scientific, Pittsburg, PA, USA). The brain was immersion-fixed overnight in periodatelysine-paraformaldehyde. After fixation, the brain was moved to PBS containing 20% glycerol and 2% dimethyl sulfoxide and stored at 4°C until sectioned. Serial coronal brain sections (40 µm) were cut on a freezing microtome with every tenth section processed as free-floating sections and stained with CD45 antibody to identify glial cells and with GFAP to identify astrocytes. The remaining series were placed in ethylene glycol cryoprotectant solution (PB solution, ethylene glycol and sucrose, pH 7.2) and stored at −20°C until used.

The staining was performed as follows. Briefly, sections were rinsed in 1 × PBS and then in PBS-Triton-X100 (Fisher Scientific). Thereafter, the sections were incubated in 0.3% hydrogen peroxide for 15 minutes to block endogenous peroxidase activity and blocked for 2hours (10% serum, 0.3% Triton). After being washed, the sections were incubated in rat anti-mouse CD45 antibody (Abdserotec, Raleigh, NC 27609) or GFAP antibody (Dako) for 3hours. The sections were then incubated with anti-rat biotinylated or anti-rabbit biotinylated, for one hour. After rinsing with PBS-Triton-X100, the sections were reacted for one hour in avid in-labeled horseradish peroxidase from an MOM kit (Vector Laboratories). The sections were incubated with 0.2 mol/L sodium acetate (pH 6.0) and differentiated with 3, 3'-diaminobenzidine (DAB, Sigma, St. Louis, MO, USA) as chromogen and nickel ammonium sulfate to intensify the reaction (Fisher Scientific), in addition to 1:100 of 30% hydrogen peroxide. The sections were washed with 0.2 mol/L sodium acetate and, thereafter, with PBS. Finally, the sections were dehydrated, cleared in Hemo-De (Scientific Safety Solvents, Keller, TX, USA), and cover slipped.

### Statistical Analysis

The Kruskal-Wallis one-way analysis of variance by rank was used to test the hypothesis that the distributions for sucrose preference ratios at baseline and on days 1, 5, 9, and 14 after surgery were equivalent. Two-tailed Student’s t-test was used to compare the sucrose preference ratios between the baseline value and the ratios on days 1, 5, 9 and 14. A p-value of less than 0.05 was considered significant. For the semi-quantitative histological analysis a Mann-Whitney one-tailed test was used. Histological analysis was compared in saline controls versus meloxicam treated mice for a given day post-splenectomy (day 5, 9 or 14). Statistical analysis was done using GraphPad Prism software, version 6.07 (GraphPad software, Inc.).

## Results

### Sucrose Preference Test

**Figure 1:**
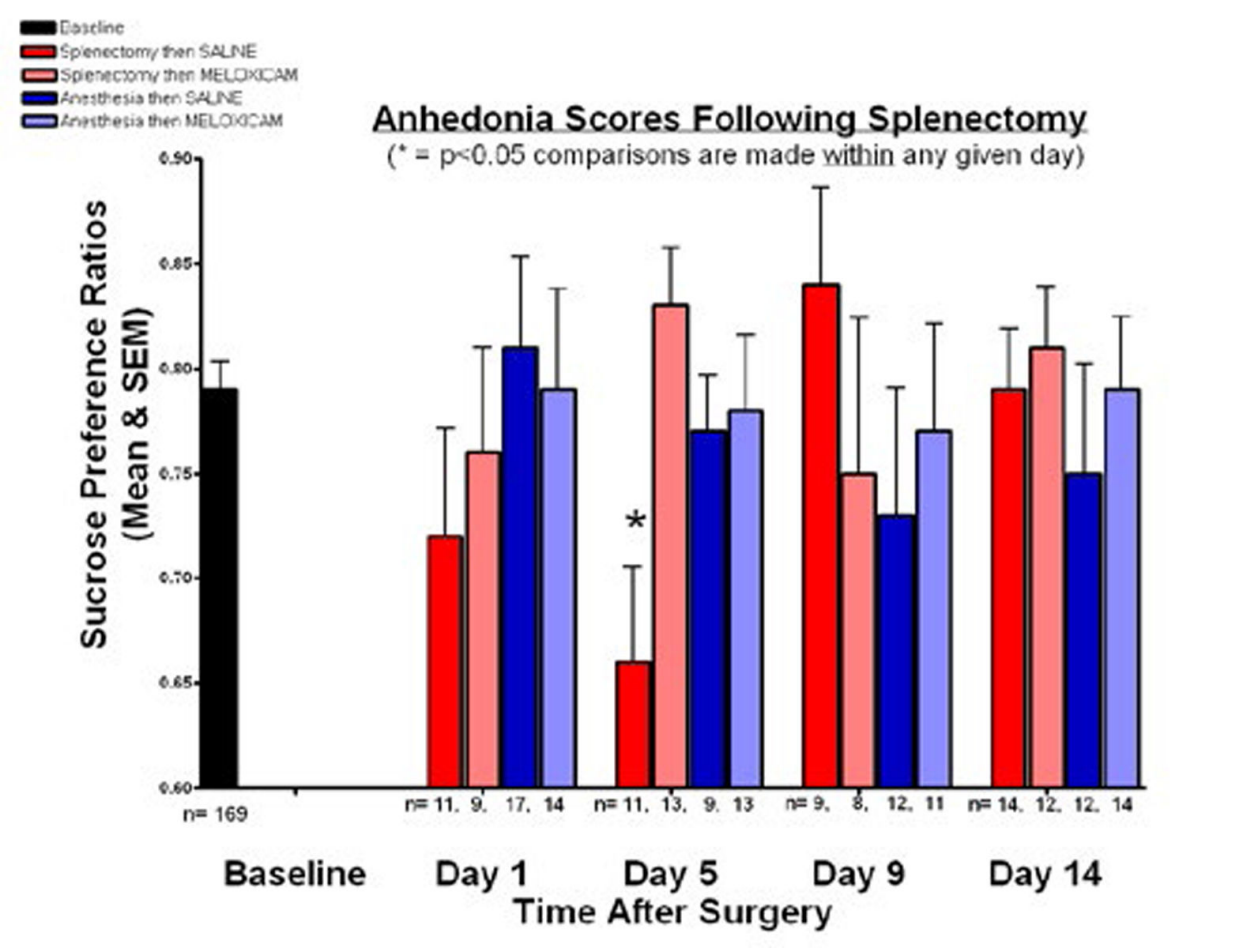
Anhedonia scores following splenectomy

The sucrose preference ratios show at baseline for all mice and for the four groups at days 1, 5, 9, and 14 after anesthesia and/ or splenectomy (Figure 1). At baseline, the mean sucrose preference ratio was 0.785 (SEM = 0.013). The baseline sucrose preference ratios were not a basis of statistical comparison because differences were sought between groups within a particular testing day.

Sucrose preference ratios were evaluated on days 1, 5, 9, and 14. There were no significant differences between groups on any day with the exception of day 5, when a significant difference between groups was found. Group 1, which received splenectomy and anesthesia followed by saline at 24 hours, had a ratio of 0.657 (SEM = 0.047) while group 2, which had splenectomy and anesthesia followed by MEL treatment, had a ratio of 0.828 (SEM = 0.029). Comparing those two groups, we rejected the null hypothesis that the sucrose preference ratios at day 5 for the splenectomy then MEL group and splenectomy then SAL group were equal (p<0.01).

Thus, MEL attenuated the surgery associated reduction in sucrose preference. Anesthesia alone, with MEL or SAL, did not change sucrose preference ratios on any day nor did surgery, with MEL or SAL, on any day besides day 5.

### Total Fluid Intake

**Figure 2:**
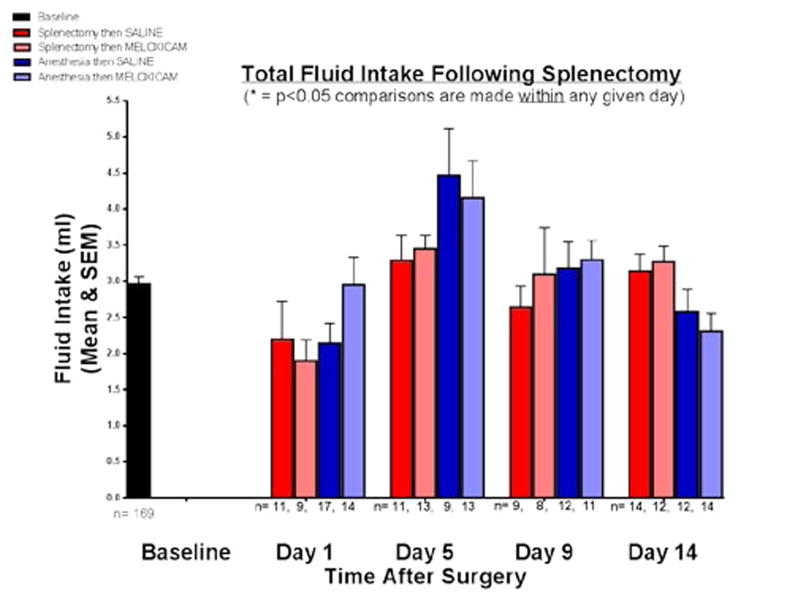
Total fluid intake following splenectomy

The total fluid intake at baseline and for the four groups on days 1, 5, 9, and 14 after splenectomy and/or anesthesia was shown in Figure 2. The mean fluid intake at baseline was 2.96 mL (SEM = 0.107 mL). At day 5, all four groups had an upward trending consumption of fluid that was not statistically significant within that day. There was no significant difference observed in the total fluid consumption between the four groups on any given day after surgery. Fluid intake, therefore, did not change sucrose preference.

### Locomotion

**Figure 3:**
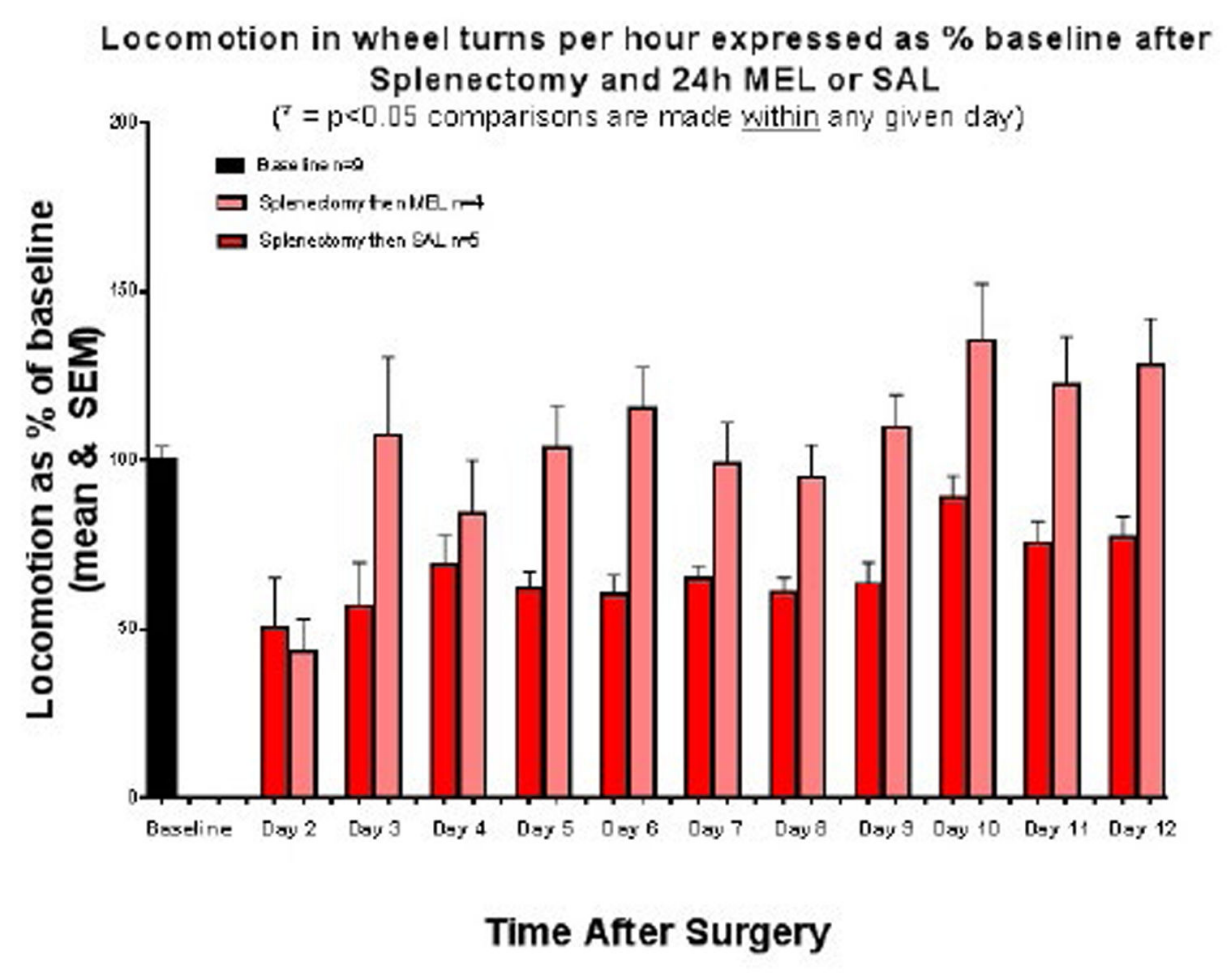
Locomotion in wheel turns per hour expressed as percentage of baseline after splenectomy and 24h MEL or SAL

Locomotion in wheel turns per hour is expressed as a percentage of baseline values (+/− SEM) after Day 0 splenectomy followed by 24 hour MEL or SAL injection (Figure 3). During the second day after injection, both groups had a similar decrease in locomotion to about 45% of baseline. By day 3, the MEL group was at baseline and remained above it until day 12. The SAL group did not return to baseline throughout the 12 day period. In addition, locomotion played no role in sucrose preference or fluid consumption.

### Immunohistochemistry

#### Astrocytosis

**Figure 4:**
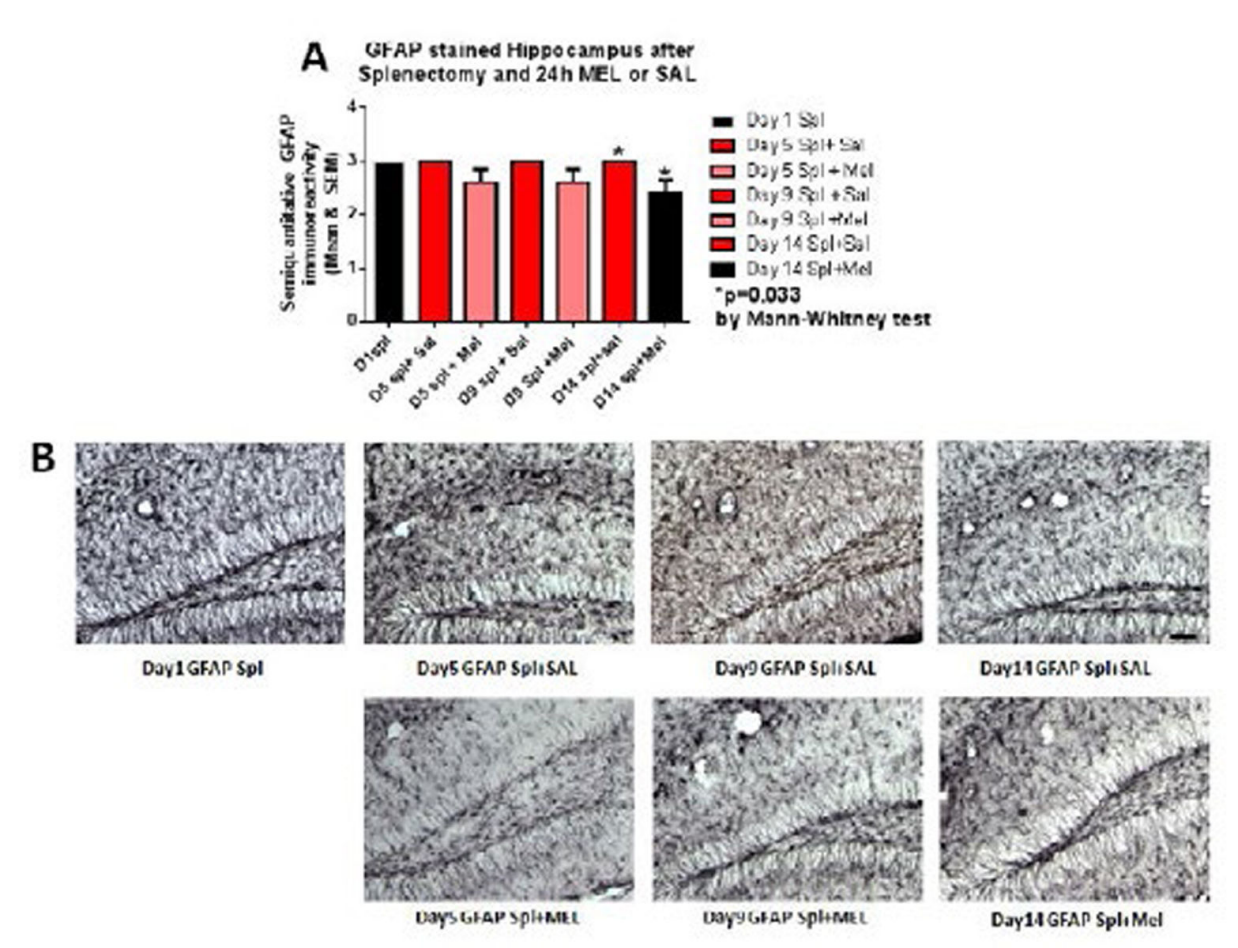
GFAP-stained hippocampus after splenectomy and 24h MEL or SAL Figure 4A is based upon ratings of semiquantitative analysis of the extent of GFAP astrocyte immunoreactivity in Figure 4B. A significant decrease of hippocampal reactive astrocytosis, as reflected by lower GFAP immunoreactivity, in mice treated with MEL after splenectomy on day 14 is seen (p=0.033 by Mann-Whitney test) (scale bar =100 µm).

**Figure 5:**
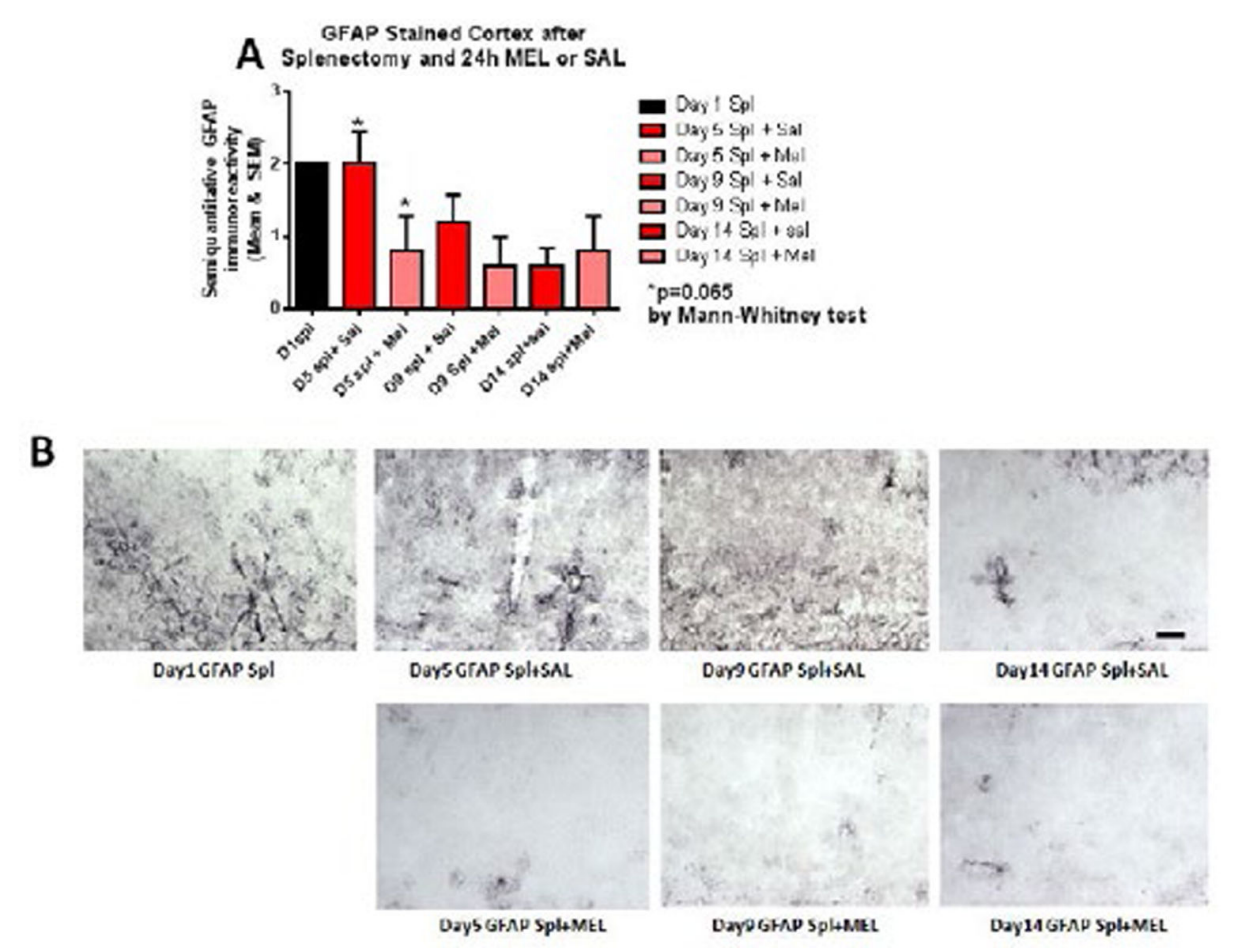
GFAP-stained cortex after splenectomy and 24h MEL or SAL Figure 5A is based upon ratings of semiquantitative analysis of the extent of GFAP astrocyte immunoreactivity in Figure 5B. A trend for reduced cortical astrocytosis is observed in mice treated with MEL after splenectomy on day 5 (p=0.065 by Mann-Whitney test). (Scale bar =100 µm).

**Figure 6:**
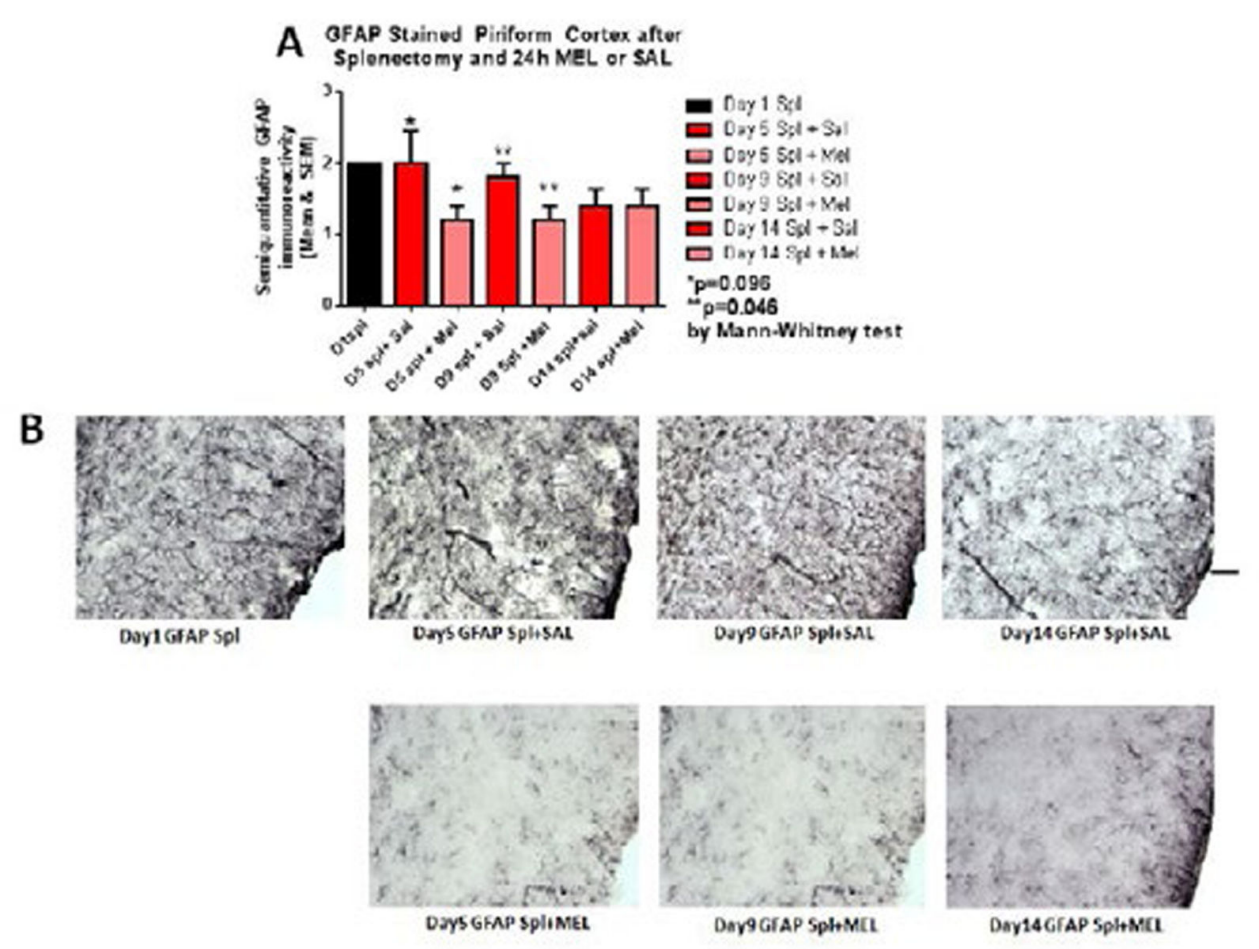
GFAP-stained piriform cortex after splenectomy and 24h MEL or SAL Figure 6A is based upon ratings of semiquantitative analysis of the extent of GFAP astrocyte immunoreactivity in Figure 6B. Decreased GFAP immunoreactivity in the piriform cortex in mice receiving MEL after splenectomy is observed on days 5 and 9 (p=0.096 and p=0.046, respectively by Mann-Whitney test)(Scale bar =100 µm).

**Figure 7:**
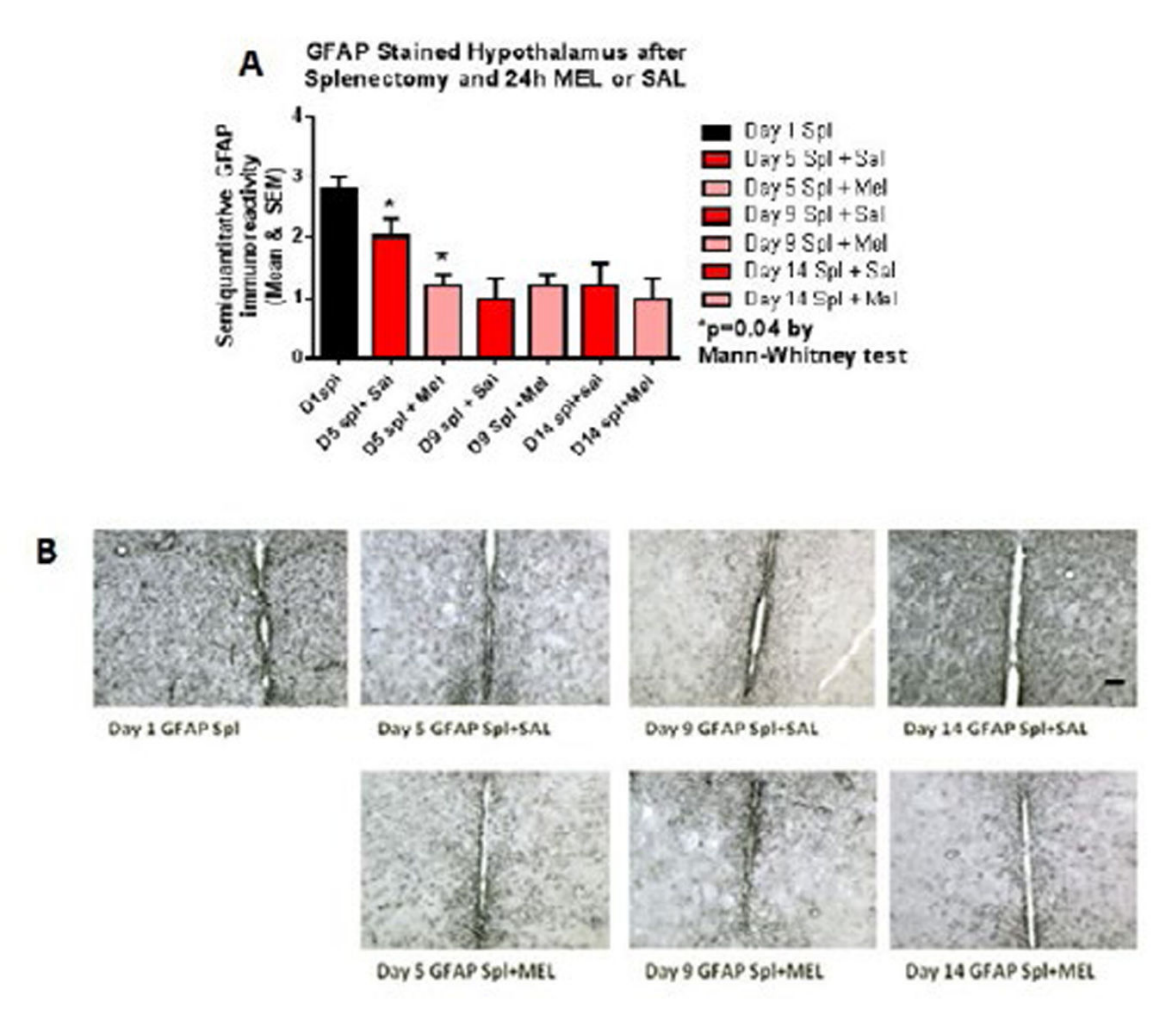
GFAP-stained hypothalamus after splenectomy and 24h MEL or SAL Figure 7A is based upon ratings of semiquantitative analysis of the extent of GFAP astrocyte immunoreactivity in Figure 7B. Decreased GFAP immunoreactivity in the hypothalamus in mice receiving MEL is observed after splenectomy on day 5 (p=0.04 by Mann-Whitney test). (Scale bar =100 µm)

Reactive astrocytosis was rated on a scale of 0 to 3. The rating was based on a semiquantitative analysis of the extent of GFAP immunoreactivity (number of GFAP immunoreactive cells and complexity of astrocytic branching), as we have previously published [12]. Semiquantitative means and SD were then determined for each group of five animals and are displayed in the graphs below. Figure 4 shows significant decrease of hippocampal reactive astrocytosis, as reflected by lower GFAP immunoreactivity, in mice treated with MEL after splenectomy on day 14 (p=0.033 by Mann-Whitney test). In Figure 5, a trend for reduced cortical astrocytosis is observed in mice treated with MEL after splenectomy on day 5 (p=0.065 by Mann-Whitney test). Figure6 demonstrates decreased GFAP immunoreactivity in the piriform cortex in mice receiving MEL after splenectomy on day 5 and 9 (p=0.096 and p=0.046, respectively by Mann-Whitney test). Figure 7 demonstrates decreased GFAP immunoreactivity in the hypothalamus in mice receiving MEL after splenectomy on day 5 (p=0.04 by Mann-Whitney test).

### Microgliosis

**Figure 8:**
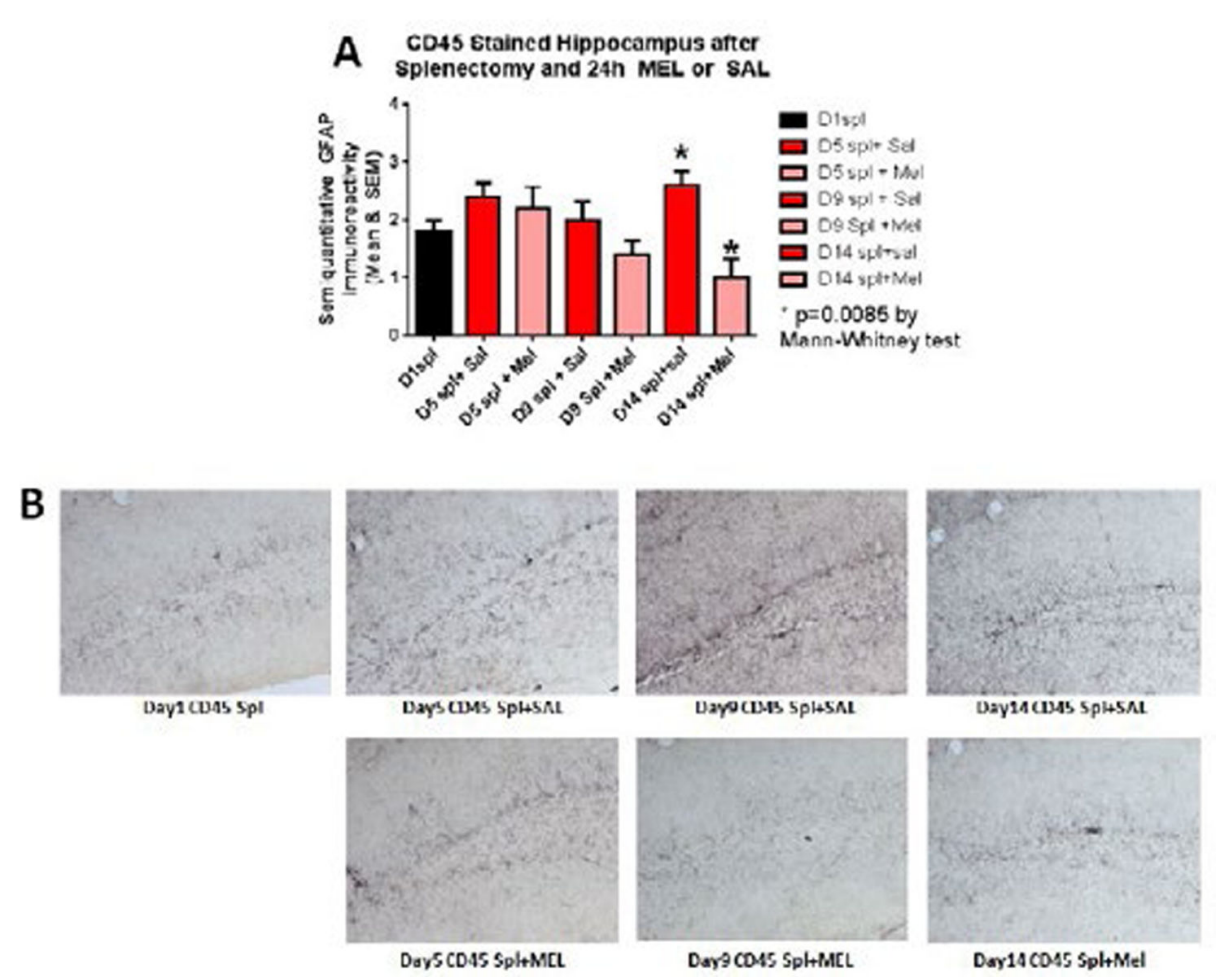
CD45-stained hippocampus after splenectomy and 24h MEL or SAL Figure 8A is based upon ratings of semiquantitative analysis of the extent of CD45 microglial immunoreactivity in Figure 8B. Decreased inflammation in the hippocampus, as measured by CD45 immunoreactivity, is observed in MEL-treated mice following splenectomy at day 14 (p=0.0085 by Mann-Whitney test). (Scale bar =100 µm)

**Figure 9:**
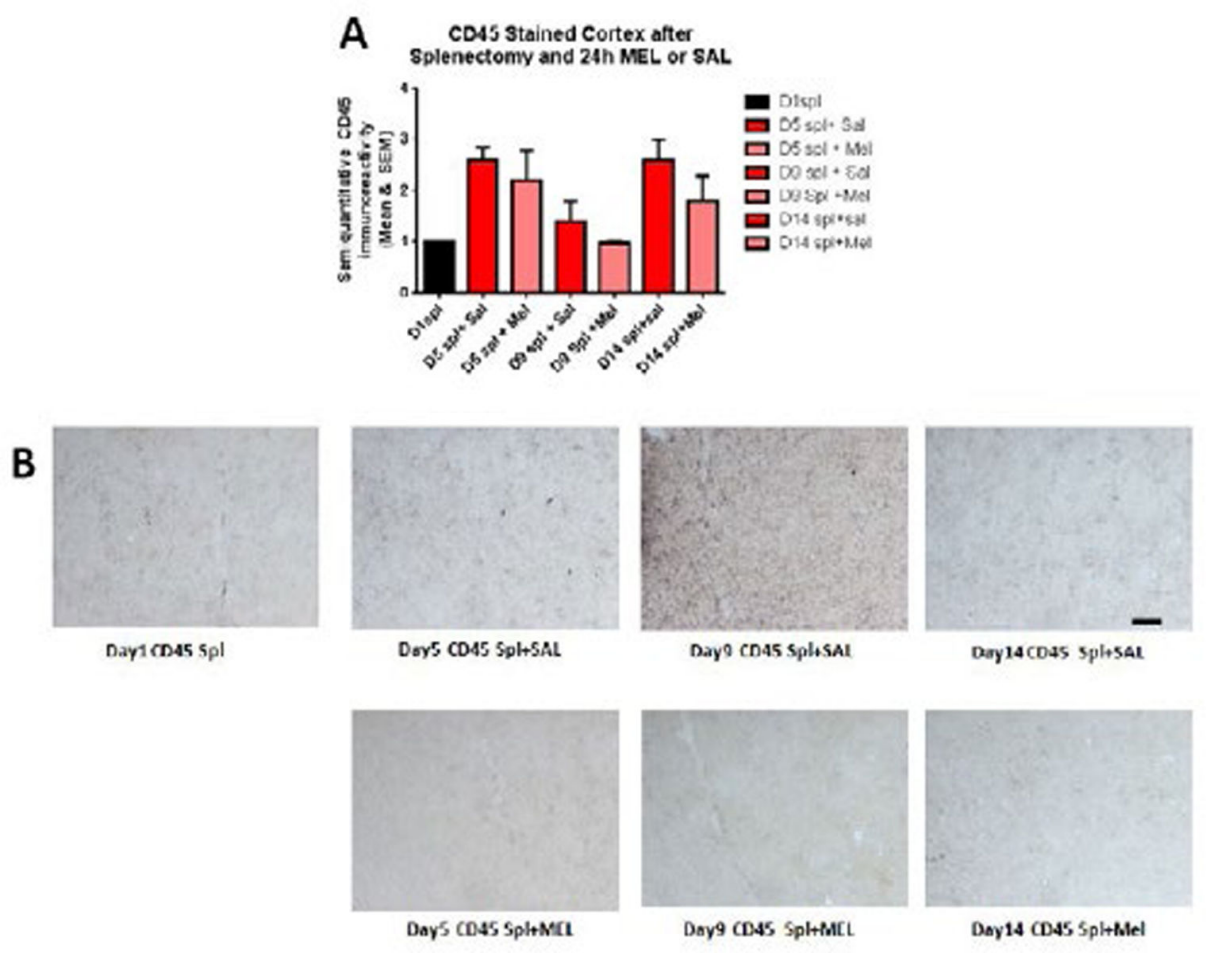
CD45-stained cortex after splenectomy and 24h MEL or SAL Figure 9A is based upon ratings of semiquantitative analysis of the extent of CD45 microglial immunoreactivity in Figure 9B. A nonsignificant trend for decreased inflammation in the cortex, as measured by CD45 immunoreactivity, is observed in MEL-treated mice following splenectomy at day 14 (Scale bar =100 µm).

**Figure 10:**
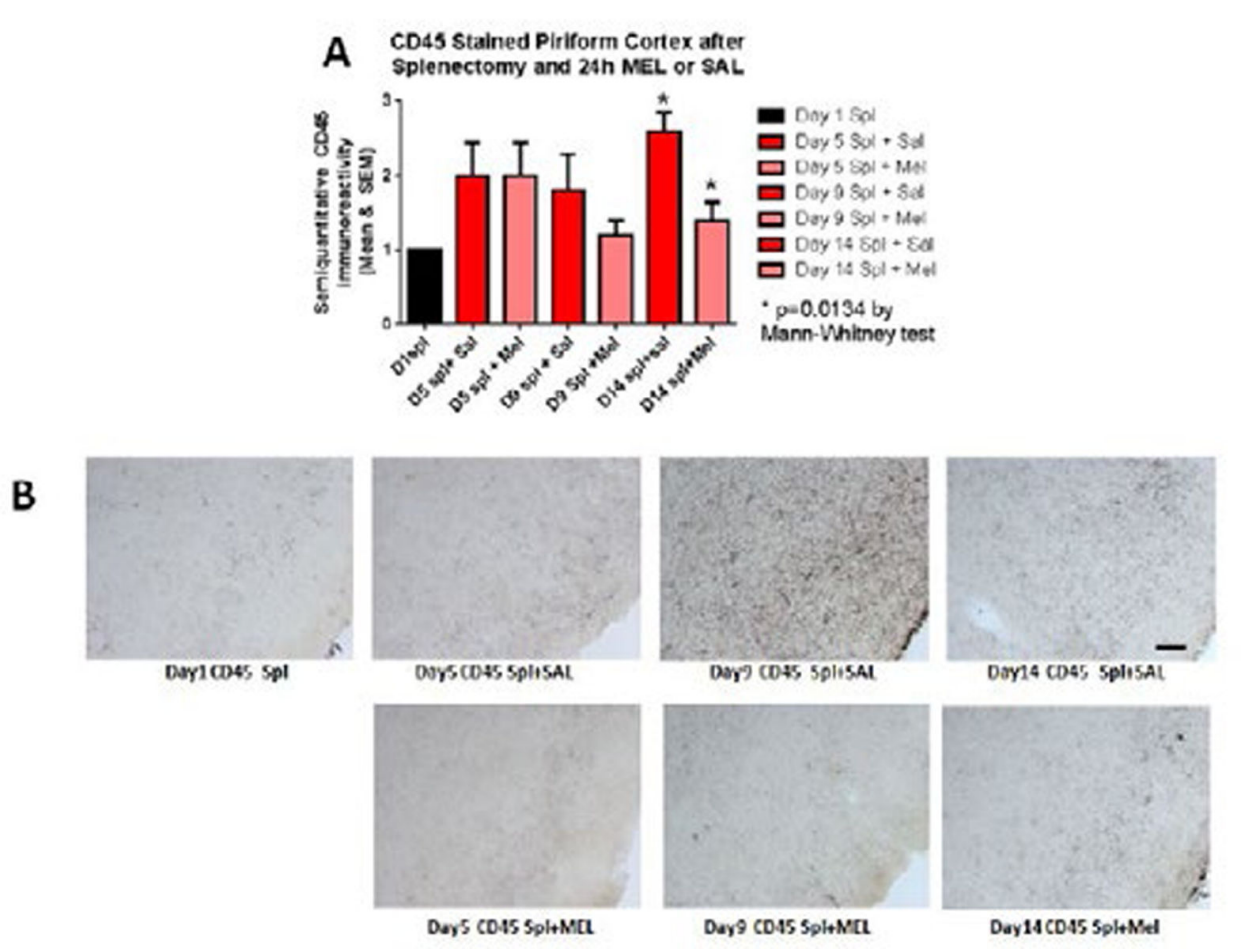
CD45-stained piriform cortex after splenectomy and 24h MEL or SAL Figure 10A is based upon ratings of semiquantitative analysis of the extent of CD45 microglial immunoreactivity in Figure 10B. Significantly reduced piriform cortical inflammation is observed on day 14 (p=0.0134 by Mann-Whitney test) in mice treated with MEL after splenectomy. (Scale bar =100 µm).

**Figure 11:**
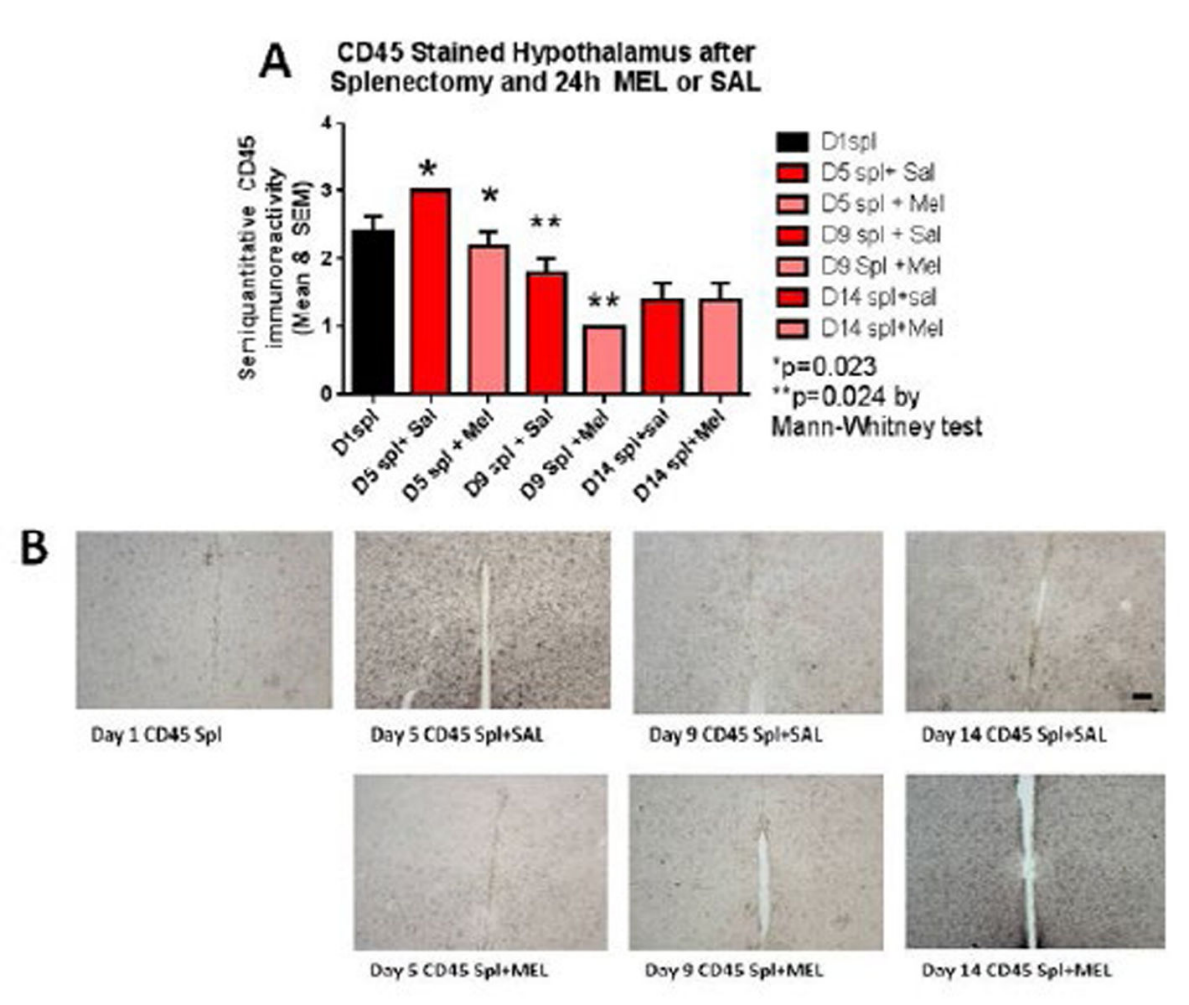
CD45-stained hypothalamus after splenectomy and 24h MEL or SAL Figure 11A is based upon ratings of semiquantitative analysis of the extent of CD45 microglial immunoreactivity in Figure 11B. Decreased CD45 immunoreactivity in the hypothalamus in MEL-treated mice is observed on days 5 and 9 (p=0.023 and p=0.024, respectively by Mann-Whitney test). (Scale bar =100 µm).

The assessment of the CD45 immunostained sections was based on a semiquantitative analysis of the extent of microgliosis (0, very few or no microglia; 1, a few ramified and/or phagocytic microglia; 2, moderate number of ramified/phagocytic microglia; 3, numerous ramified/phagocytic microglia), as we have previously reported [10]. Semiquantitative means and SD were then determined for each group of five animals and are displayed in the graphs below. In Figure 8 significantly decreased inflammation in the hippocampus, as measured by CD45 immunoreactivity, is observed in MEL-treated mice following splenectomy at day 14 (p=0.0085 by Mann-Whitney test). In Figure 9a trend for decreased inflammation in the cortex, as measured by CD45 immunoreactivity, is observed in MEL-treated mice following splenectomy at day 14. Figure 10 shows significantly reduced piriform cortical inflammation on day 14 (p=0.0134 by Mann-Whitney test) in mice treated with MEL after splenectomy. Figure 11 demonstrates the trend of decreased CD45 immunoreactivity in the hypothalamus in MEL-treated mice on days 5 and 9 (p=0.023 and p=0.024, respectively by Mann-Whitney test).

## Discussion

This study found that splenectomy in mice is associated with-anhedonia and neuroinflammation evidenced by behavioral changes, microgliosis, and astrocytosis. In addition, this study supports our additional hypothesis that postsurgical treatment with the COX-2 inhibitor MEL would attenuate both anhedonia and neuroinflammation. Furthermore, anesthesia alone with or without MEL treatment had no effect on mood. These findings are supportive of our previous work where MEL attenuated short-term memory loss and hippocampal microglial activation following splenectomy in mice. Surgery anatomically distant from the CNS produced the combined sequelae of mood and memory changes with associated neuroinflammation that an anti-inflammatory treatment was able to modulate.

Comparisons for sucrose preference were made between treatment groups within a given test day rather than in comparison to the baseline measurements. Diminished sucrose preference indicative of anhedonia can be reversed by tricyclic antidepressants and can be used as an analogue for depressive behavior in mice [10,17]. On day 1 after splenectomy, anhedonia was not observed in either control mice with saline or MEL-treated mice. However at day 5, mice treated with SAL experienced decreased sucrose preference, reflecting anhedonia, while mice treated with MEL did not exhibit anhedonia. Anhedonia was not observed in either control with Saline or MEL-treated mice at days 9 and 14. Mice receiving anesthesia alone followed by either SAL or MEL did not exhibit decreased sucrose preference at any stage, suggesting that the administration of anesthesia is not the cause of the behavioral changes observed. The anesthetic procedures used here were comparable to those of Cao et al.[18], wherein anesthesia was also shown to have no role in the development of cognitive dysfunction.

Comparisons of reactive astrocytosis in treated mice versus control showed significant decreases on days 5, 9 and/or 14 depending on the region examined by the Mann-Whitney test for non-parametric data. Thus, we found a temporal association between hypothalamic inflammation and anhedonia as well as the absence of anhedonia after COX-2 treatment. Hippocampal CD45 microglia staining showed a significance decrease on day 14.We did not see the same pattern of microglial activation as in our previous work using tomato lectin antibodies, where activation peaked at day 5 and declined thereafter [10]. This may be because of the different histological methods used. The hippocampus is very prone to dysfunction following the activation of the immune system [19]. We observed that overall neuroinflammation decreased in MEL treated animals versus SAL treated animals. Significant difference was also observed in CD45 between MEL and SAL treated animals in all the pyriform cortex and hypothalamus. This is consistent with other reports that show meloxicam can reduce hippocampal inflammatory gene expression in settings such as HIV infection [20].

Overall, in GFAP-stained sections, a significant difference was observed in hippocampus, cortex piriform cortex and hypothalamus; although, the day(s) these reductions were observed were distinct in different brain regions. The interaction between microglia and astrocytes, which produce neurotrophins during neuroinflammation is not clear [21,22]. Astrocytes support and sustain the CNS milieu but are increasingly thought to play a role in the inflammatory response [23,24]. However, in this study, microglial activation has a stronger association with anhedonia than does astrocytosis. Hypothalamic inflammation secondary to cytokines can lead to depression via the Hypothalamus-Pituitary-Adrenal (HPA) axis by deregulating the activity of the sympathetic nervous system and the endocrine release of stress hormones and glucocorticoids [25]. Peripheral inflammation secondary to cytokines causes release of free radicals, oxidants, and glucocorticoids that change microglial cell function and may damage neurons. These findings are in keeping with the putative greater role of microglia in neuroinflammation.

While all groups of mice exhibited an upward trend in total fluid consumption on day 5 regardless of treatment after either splenectomy or anesthesia alone, there was no significant difference in fluid intake between groups on any given day. This, in addition to the alternating of tube position, indicates that neither thirst nor water or sucrose tube placement were factors in the behaviors observed. Locomotor testing showed that the treatment used after splenectomy resulted in different behaviors over a 12 day period, with MEL-treated mice significantly more active throughout. Therefore, locomotion played no role in sucrose preference or fluid consumption. It could be argued that our locomotor and anhedonia findings are caused by pain. Pain, one of the cardinal manifestations of trauma and inflammation, can also result in decreased motor activity that mirrors that of depressive behavior [26]. In addition, cytokine-associated hyperalgesia has been observed in several different models [27,28]. The potential effects of pain on movement are obvious, and concurrent pain may change motivation to counter thirst in a particular manner. However, all of the above findings in concert show a MEL effect long past its reported half-life. Oral or intravenous treatment of mice with MEL shows a half-life of 4.8 and 6.4 hours, respectively, with the i.p. half-life likely to be intermediate. MEL is metabolically cleared with 8% of the dose remaining at 72 hours, followed by complete elimination at 96 hours in mice [29]. Therefore, a direct effect of MEL on pain is unlikely when behavioral changes are observed at day 5 and, with regards to locomotion, through day 12.

Furthermore, in a model of acute hypotension, a similar transient delayed pattern of neuroinflammation, memory loss, and anhedonia was noted by our group. We found that MEL given either immediately after or eight hours after surgery had no significant effect on ameliorating cognitive dysfunction, but there was a significant reduction in cognitive dysfunction when MEL was given 24 hours following surgery[10]. This observation corresponds to the finding that cytokine release peaks at 24 hours following brain damage in rodents [30]. Our timing of MEL treatment was based on a likely COX-2 based effect of MEL on cytokine function in the immediate period after 24 hours rather than a longer lasting direct effect of the drug.

The cytokine-mediated inflammatory response following surgical trauma, therefore, is the likely target of COX-2 inhibition in our study. This is similar to the response elicited by infection or injury, and can present with comparable manifestations. Postoperative inflammation is associated with cognitive dysfunction and depression [1–4]. Peripheral immune stimulation in response to infection or injury stimulates the release of cytokines that may change behavioral and motivational states. These changes, which may manifest as anorexia, malaise, lethargy, decreased activity, and social withdrawal, reorganize the individual’s priorities towards fighting infection or healing from injury [30–32]. Such changes may simultaneously serve as an evolutionary adaptive behavior to minimize the physical or psychological impact of a stressor and to facilitate responses to help overcome it [33,34]. Collectively, this constellation of changes is referred to as “sickness behavior.” Just like the fear response that is provoked by the threat of a predator, sickness behavior is an adaptive and normal biologic response that can become pathologic if it arises in the wrong context or is excessive in intensity or duration.

Because sickness behavior and major depressive disorder share many similar clinical manifestations, it has been postulated that major depressive disorder represents a maladaptive pattern of sickness behavior that has been inappropriately activated or out of proportion to what would be expected [35,36]. Studies have shown that while sickness behavior tends to manifest relatively early on in the time course following cytokine treatment before dissipating within 24 hours, depressive behavior has a more delayed onset and persists beyond 24 hours [37,38]. These findings suggest that despite their similar clinical manifestations, sickness behavior and depression are two distinct entities. Immunological challenges may also provoke the development of depression, and psychological stress, in turn, can compromise immune function [34]. Such stressors, which can include discrete negative life events (e.g. bereavement, loss of a job) or as the culmination of many negative events (e.g. daily struggles of life) [39], activate the HPA axis and can dysregulate the synthesis and activity of neurotransmitters such as serotonin, norepinephrine, and dopamine [34].

The interplay between stress and processes involving the endocrine and immune systems suggests a role for inflammation in the development of depression. Indeed, depression has been described as an inflammatory disease characterized by elevated levels of cytokines [40–42]. Major depressive disorders have been reported in patients being treated with the recombinant cytokines interleukin-2 (IL-2) and interferon-α (IFN-α) [43], and chronic inflammatory diseases such as coronary artery and autoimmune diseases have been associated with an increased incidence of mood disorders [37]. Other studies have shown that IL-1β-treated rats have decreased performance in a task required to obtain a sucrose solution reward, and rats given lipopolysaccharide (LPS), a potent inducer of cytokine production, experienced persistent reduction in motor activity and a decreased preference for a sweetened drinking solution [38,45]. Cytokines in the periphery activate the transcription of cyclooxygenase-2(COX-2), which synthesizes prostaglandins vital to the cardinal signs of inflammation and to sustaining the inflammatory response. In addition, acute peripheral inflammation activates the brain’s innate immune system (i.e. microglia), central nervous system expression of cytokines, and other inflammatory mediators. Therefore, COX-2 function has a role in postsurgical neuroinflammation that contributes to cognitive dysfunction and possibly to depression [46–48].

Peripheral cytokines can stimulate the release of other proinflammatory cytokines and activate the transcription of COX-2, which is the rate-limiting enzyme in the production of prostaglandins and is suspected of playing a role in the postsurgical neuroinflammation [46–48]. Cytokines act centrally by altering the permeability of the Blood-Brain Barrier (BBB) via increased expression of vasoactive and inflammatory molecules such as COX-2 and histamine [47]. Endothelial cells of the BBB have shown up-regulated transcription of nuclear factor kappa B (NF-κB), a signal transduction protein shared by many cytokines, and increased release of prostaglandins and inflammatory mediators like COX-2 and histamine in response to cytokines [47]. It has been proposed that cytokine activation in the periphery triggers neuroinflammation by either a humoral or neuronal pathway, modulated by upregulation of a number of peripheral inflammatory markers [48,49]. The resulting changes to the brain can manifest as fever, anorexia, and cognitive impairment.

Our study has several limitations. One is the possible role of pain in our findings, which we have discussed above. Another potential limitation is the possibility that while anhedonia may be indicative of a mood disorder, it is also a symptom of cytokine-mediated sickness behavior. This is also discussed above. It is likely that sickness behavior-associated mood changes are on a temporal and pathological continuum with depression despite their different time courses.

In summary, we have shown that postoperative anhedonia and neuroinflammation develop in a delayed transient manner following splenectomy in mice and are attenuated by delayed MEL treatment. These findings suggest that COX-2-dependent mechanisms may play a role in the development of postoperative mood disorders, possibly via modulation of peripheral effects on CNS neuroinflammation. The results indicate that future research is warranted to further elucidate the role of COX-2-dependent or other immune modulator effects on the onset of postoperative sickness behavior and mood disorders-in translational and clinical studies.
